# Causes and consequences of cytonuclear incompatibility in hybrids of flowering plants

**DOI:** 10.1093/jxb/erag075

**Published:** 2026-02-12

**Authors:** Mehrdad Shahbazi, Joel Sharbrough, Jana Knerova, Jonathan F Wendel, David Kopecky

**Affiliations:** Institute of Experimental Botany of the Czech Academy of Sciences, Centre of Plant Structural and Functional Genomics, Šlechtitelů 31, Olomouc 77900, Czech Republic; National Centre for Biomolecular Research, Faculty of Science, Masaryk University, Kotlářská 2, Brno 61137, Czech Republic; Department of Ecology, Evolution, and Marine Biology, University of California, Santa Barbara 93106, CA, USA; Institute of Experimental Botany of the Czech Academy of Sciences, Centre of Plant Structural and Functional Genomics, Šlechtitelů 31, Olomouc 77900, Czech Republic; Department of Ecology, Evolution, and Organismal Biology, Iowa State University, Ames 50011, IA, USA; Institute of Experimental Botany of the Czech Academy of Sciences, Centre of Plant Structural and Functional Genomics, Šlechtitelů 31, Olomouc 77900, Czech Republic; University of Glasgow, UK

**Keywords:** Allopolyploidy, chloroplast, cytonuclear interactions, gene expression, interspecific hybridization, mitochondria, organellar DNA, trafficking, whole genome duplication

## Abstract

Hybridization and polyploidization combine divergent nuclear genomes with maternally inherited organelles, often disrupting cytonuclear coadaptation critical for respiration and photosynthesis. This review examines the mechanisms, outcomes, and evolutionary significance of cytonuclear incompatibility in plants. We focus on how divergence in nuclear-encoded, organelle-targeted proteins and organelle genomes leads to mismatched interactions in protein import, folding, and assembly of multi-subunit enzyme complexes. The evidence highlights taxon- and complex-specific responses that mitigate incompatibilities, including the biased retention and expression of maternal alleles, gene conversions, and regulatory adjustments. We highlight how cytonuclear compatibility in hybrid lineages entails responses at multiple levels of regulation, including DNA methylation/chromatin accessibility, gene expression, alternative splicing, translation rates, organelle import, protein-folding and assembly, and protein degradation pathways. Manifestations such as chlorosis, seed sterility, or hybrid breakdown underscore the role of cytonuclear incompatibilities in shaping reproductive barriers. Conversely, maternal bias and compensatory mechanisms often act to restore functional integration of parental genomes, allowing hybrid and polyploid persistence. Beyond their evolutionary role in speciation and adaptation, cytonuclear incompatibilities underpin key practical applications, notably cytoplasmic male sterility, a cornerstone of hybrid crop breeding. We conclude that cytonuclear dynamics reveal both constraints and opportunities, illuminating plant diversification, hybrid resilience, and agricultural innovation.

## Teaser

This review synthesizes our understanding of the mechanisms of cytonuclear incompatibility in plant hybrids and allopolyploids, highlighting mechanisms underlying maternal bias, evolutionary consequences, and its practical applications in seed production.

## Introduction

Mitochondria and chloroplasts are key components of a plant cell, responsible for the two most important energy-processing functions, photosynthesis and cellular respiration. Mitochondria and chloroplasts were derived from free-living α-proteobacteria ∼2 billion years ago and from cyanobacteria ∼1.5 billion years ago, respectively (Andersson *et al*., 1998; Martin *et al*., 2002). Their genomes are highly reduced compared to their free-living progenitors, having achieved this reduction either by outright gene loss or by gene transfer to the host genome (Gray *et al*., 1999; Timmis *et al*., 2004). Such transfer of organellar genes significantly affected not only the size of the organellar but also the nuclear genome. Some 18% of the nuclear genome in *Arabidopsis thaliana* (Brassicaceae) is of cyanobacterial (i.e. chloroplast) origin (Martin *et al*., 2002).

Both mitochondria and chloroplasts perform protein synthesis on their own ribosomes. However, their genomes lack the full (animals) or partial (plants) complement of genes for the ribosomal proteins (Boore, 1999; Adams, 2003). Therefore, the assembly of functional organellar ribosomes and organellar protein synthesis requires import of nuclear-encoded proteins that were originally encoded in the organellar genomes but were transferred to the nuclear genome. Similarly, many genes required for the synthesis of proteins involved in complexes responsible for photosynthesis and cellular respiration were transferred to the nucleus during the evolution of organelles (Millar *et al*., 2007; Van Wijk and Baginsky, 2011). It is estimated that ∼2000 and ∼3000 proteins that comprise mitochondrial and plastid proteomes, respectively, are nucleus-encoded (Forsythe *et al*., 2019). Many protein complexes, including RuBisCO (Ribulose-1,5-bisphosphate carboxylase/oxygenase) in chloroplasts and the OXPHOS (oxidative phosphorylation) complexes in mitochondria, are composed of proteins encoded by both nuclear (organelle-targeted) and organellar genes. Thus, both nuclear and organellar components involved in cytonuclear protein complexes were subjected to the co-evolutionary requirements of co-residency in the same organelle. Such co-evolution by reciprocal changes in interacting proteins has been reported in many species (reviewed in (Rand *et al*., 2004); Sloan *et al*., 2018).

The nuclear-encoded proteins involved in cytonuclear complexes navigate from the nucleus to the organelles. In the case of RuBisCO, the nuclear gene *RBCS* (nuclear gene encoding small subunit of RuBisCO) is transcribed into messenger RNA (mRNA), which is transferred to the cytoplasm and translated on ribosomes into the SSU (small subunit of RuBisCO) protein (Nierzwicki-Bauer *et al*., 1984). At the N-terminus of the SSU protein is a transit peptide (a short amino acid sequence), which acts as a signal directing the protein to the chloroplast. The protein is recognized by receptors on the outer chloroplast membrane. These receptors directly interact with protein import machinery including the TOC (translocon at the outer chloroplast membrane) and TIC (translocon at the inner chloroplast membrane) complexes (Schnell *et al*., 1997). The SSU protein is threaded through the TOC/TIC complexes and translocated into the stroma, the fluid-filled space within the chloroplast. Once inside the stroma, the transit peptide is cleaved by a specific protease and the SSU protein associates with the chloroplast-encoded large subunit (LSU; encoded by *RBCL*; organellar gene encoding large subunit of RuBisCO) to assemble the functional RuBisCO enzyme (reviewed in Parry *et al*., 2007; Sharkey, 2023). In essence, the nucleus provides the blueprint for SSU, but the protein needs to be directed and translocated into the chloroplast for proper function. This process involves a sophisticated interplay between the nuclear-encoded protein, the import machinery of chloroplasts, and the transit peptide. Thus, it is evident that the co-evolution, and potentially coadaptation, precisely tuned the compatibility of nuclear and organellar partners of protein complexes. However, this compatibility can be disrupted by the presence of divergent variants of the nuclear genes in interspecific hybrids and allopolyploids (Sharbrough *et al*., 2017). Because most eukaryotes inherit organelles maternally (Camus *et al*., 2022), the variant of nucleus-encoded protein(s) from the paternal genome may not be fully compatible with the maternal organelle-encoded protein(s) in interspecific hybrids (Birky, 1995; Greiner *et al*., 2015).

### Interspecific hybridization and cytonuclear interactions

The process of hybridization entails the merger of two distinct nuclear genomes with the cytoplasmic genomes originating from a single parent (usually the maternal parent; Camus *et al*., 2022). If the maternally derived half of the nuclear genome has co-evolved with the cytoplasmic genomes, the hybrid may exhibit reduced photosynthetic or respiratory performance due to mismatches between the gene products encoded by the paternally derived subgenome and genes or gene products from the cytoplasmic genomes (Rand *et al*., 2004; Sloan *et al*., 2018). The potential for such cytonuclear incompatibilities to negatively affect function is particularly likely in diploid F_2_ hybrids (Burton *et al*., 2013), wherein the F_2_ hybrid can, by segregation alone, lose the maternally derived compatible nuclear alleles (Barreto *et al*., 2018). Additionally, there may be functionally important differences (e.g. amino acid changes at contact residues) between the cytoplasmic genomes of parental species, as in the case of cytoplasmic male sterility phenotypes (Chase, 2007). In most plant species, the rate of sequence evolution in plastid and mitochondrial genomes is much lower than that of the nuclear genome (Sloan, 2015), but, in principle, changes in these organelles may affect cytonuclear integration. Allopolyploids are further buffered from such dynamics because both the maternal and paternal genomes are stably inherited, such that hybrid breakdown is delayed or altogether averted (depending upon the frequency of homoeologous pairing at meiosis).

The phylogenetic distance between the parental species and the functional discordance of the parental forms of the genes/proteins involved in cytonuclear complexes appears to positively correlate with the disruption in the protein-protein interactions in interspecific hybrids and cybrids (organisms having nuclear genome from one species and organellar genome from the other species; (Špírek *et al*., 2000; Barnard-Kubow *et al*., 2017; Abdel-Ghany *et al*., 2022). The extreme case is cytonuclear incompatibility, a specific type of Bateson-Dobzhansky-Muller (BDM) genetic incompatibility caused by improper and nonfunctional interactions between organellar and nuclear genomes (Chou and Leu, 2010). BDM incompatibilities can cause inviability or infertility of the interspecific hybrids leading to reproductive isolation between species/populations (Sloan *et al*., 2017). The organellar and nuclear genes involved in cytonuclear interactions are among the potential candidates responsible for this phenomenon, as strong reproductive isolation can be created by only a few interacting genes. Indeed, cytonuclear incompatibility has been observed in a variety of organisms, from primates, amphibians, and insects to plants (Liepins and Hennen, 1977; Barrientos *et al*., 1998; Chase, 2007; Ellison *et al*., 2008).

**Fig. 1.**
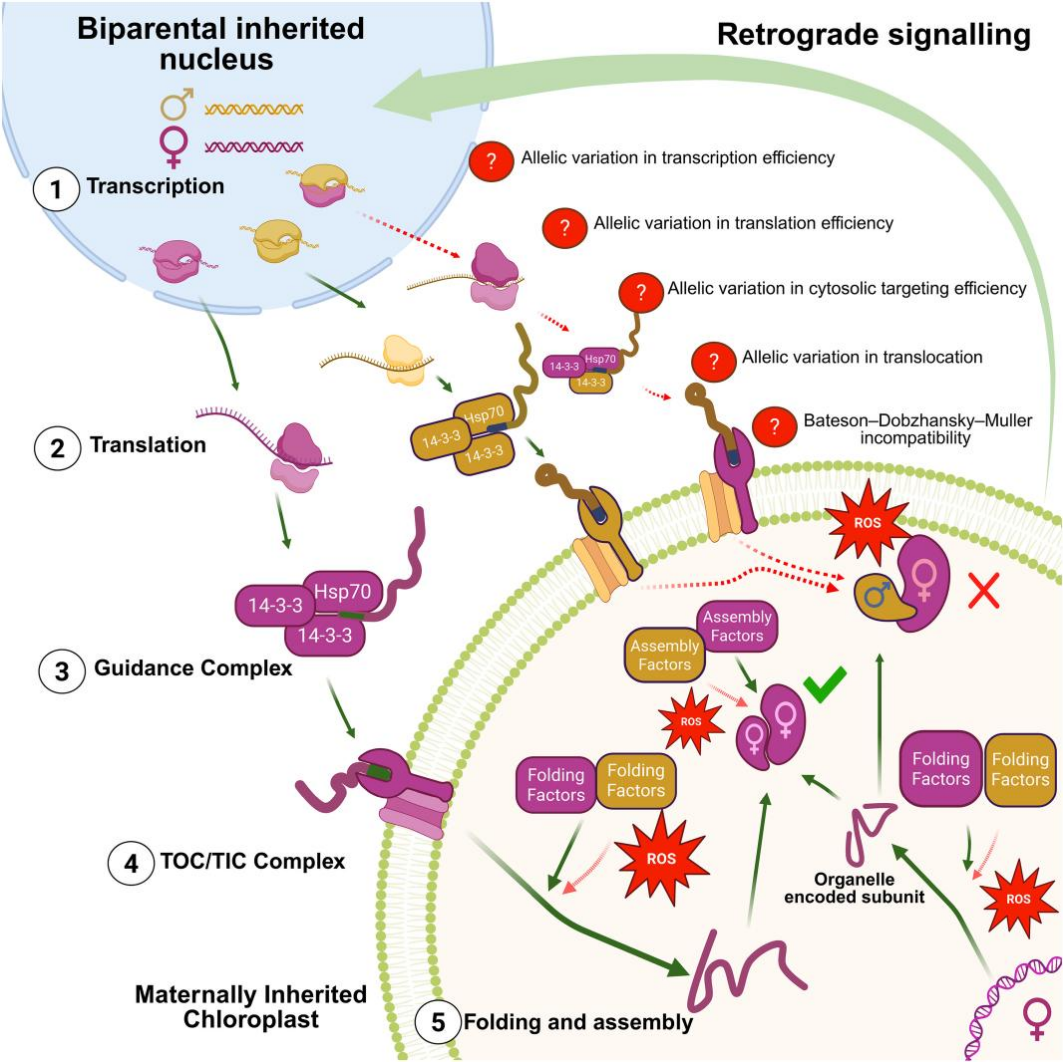
Cytonuclear coordination and potential incompatibilities in plant hybrids. In interspecific hybrids, nuclear alleles inherited from both paternal (gold) and maternal (magenta) genomes undergo nuclear transcription (1), producing transcripts that are translated into precursor proteins in the cytosol (2). These precursors are guided by chaperones such as Hsp70 and 14-3-3 proteins (3) toward plastids. During protein import across the chloroplast membrane via TOC/TIC (Translocon of the Outer/Inner Chloroplast membrane) complexes (4), paternal allelic variants may encounter problems if their targeting sequences or folding requirements differ from the maternal chloroplast system. Inside the maternally inherited chloroplasts, imported nuclear-encoded subunits must assemble with plastid-encoded partners into functional complexes (5). Arrows represent distinct functional outcomes. Solid dark green arrows indicate the normal and efficient flow of transcription, translation, targeting, and successful protein assembly. Red dotted arrows mark low-quality or inefficient processes, such as weak targeting, defective import, or improper assembly. Large green arrow shows retrograde signaling from the chloroplast to the nucleus, usually triggered by stress or incompatibility. The site marked with a red X denotes a failure point where paternal proteins are rejected or destabilized, while red bursts indicate the accumulation of reactive oxygen species (ROS) caused by misfolded or incompatible proteins. Incompatibilities may arise through interactions between paternal nuclear-encoded proteins and maternal plastid components, leading to misfolding, impaired assembly, and/or ROS production. By contrast, maternally derived nuclear alleles likely remain compatible with plastid machinery, ensuring proper folding and assembly of organelle complexes. Red circles with question marks represent the potential triggers of distorted functionality of cytonuclear complexes. Created in BioRender. https://BioRender.com/xpvte5t.

The presence and expression of divergent parental variants of nuclear genes might affect cytonuclear complexes in interspecific hybrids in different ways: during transcription, translation, trafficking from cytoplasm to organelles via TOC/TIC complexes, protein folding with the involvement of chaperonins, and assembly of the cytonuclear complexes assisted by chaperones (both chaperones and chaperonins are nuclear-encoded) (Fig. 1). In principle, the problem of cytonuclear incompatibility can be solved by various mechanisms, including the elimination, replacement, down-regulation or silencing of the incompatible paternal allele(s), , and preferential import and assembly of maternal proteins (Sloan *et al*., 2024).

Here, we review each of the aforementioned molecular processes involved in cytonuclear compatibility and interaction, where the divergence of orthologs (variants of genes from divergent parents in interspecific hybrids) can potentially cause cytonuclear incompatibility. We also outline the ways by which compatibility can be restored, and thus ensure viability and fertility of the hybrids, with obvious implications for speciation and interspecific introgression. Considering the central role hybridization has played in the domestication of many of our most important crops, we also discuss the potential challenges and opportunities cytonuclear incompatibilities present for plant breeding efforts. In total, we aim to provide access to a burgeoning literature on cytonuclear coevolution and the resulting consequences for hybrid lineages, and to provide a prospective view on paths to progress in understanding the challenges hybrid lineages face and solve.

### Pitfalls in cytonuclear incompatibility studies

The main constraint in studies on cytonuclear incompatibilities is cytonuclear incompatibility itself. Once the incompatibility in the developing F_1_ hybrid, either homoploid or allopolyploid, is strong enough to cause non-viability or sterility, there is no opportunity to assess and analyze the dynamics of cytonuclear interactions in subsequent generations. Even individuals with reduced fertility due to these incompatibilities are unlikely to be evolutionary successful. Thus, we can only study the ‘winners’, the individuals in which fertility is not compromised and, therefore, the cytonuclear incompatibilities are lessened or nonexistent. These factors may explain the inconsistency of results from studies on the extent of selection pressure on cytonuclear interactions (Ferreira De Carvalho *et al*., 2019; Grover *et al*., 2022; Shahbazi *et al*., 2024). For this reason, the use of newly developed synthetic hybrids, whose establishment, development, and propagation can be manipulated, provide useful models for such studies.

### Consequences of cytonuclear incompatibilities in hybrids of flowering plants

The most common consequence of cytonuclear incompatibilities is impaired organelle function, such as reduced photosynthesis and respiration rates, that affect fitness-related traits. Leaf pigmentation anomalies, chlorosis, and variegation are associated with reduced photosynthetic capacity and malfunction of photosystem II and have commonly been reported in interspecific hybrids. More severe phenotypes include embryonic lethality, reduced fertility, and inviability (reviewed in (Greiner *et al*., 2011; Greiner and Bock, 2013). F_1_ hybrids of wild *Pisum sativum* subsp. *elatius* (Fabaceae) with cultivated pea lines are almost completely sterile with chlorophyll deficiency and variegation when the wild pea is the female parent. The involvement of cytonuclear incompatibilities is indicated by fully viable and fertile hybrids from reciprocal crosses (Bogdanova *et al*., 2009). Hybrids of *Atropa bella-donna* (Solanaceae) with tobacco (*Nicotiana tabacum*; Solanaceae) show chlorophyll deficiency, producing an albino phenotype. This is a result of incorrect RNA editing of organelle genes. Many organelle transcripts require RNA editing, usually involving C to U conversion. However, editing sites were highly species-specific (Schmitz-Linneweber *et al*., 2005).

Mismatched nuclear and mitochondrial genomes in interspecific hybrids typically result in the disruption of anther development and the production of non-viable pollen. This phenomenon, one of several that can lead to cytoplasmic male sterility (CMS; reviewed in (Horn *et al*., 2014; Bhattacharya *et al*., 2024; Bohra *et al*., 2025)) is underlined by the disruption of the interaction between mitochondrial genes, causing aborted pollen development while nuclear male fertility restorer (*Rf*) genes prevent this (Sloan *et al*., 2024).

In natural populations, cytonuclear incompatibilities have been shown to play an important role in the formation of hybridization barriers and speciation (Chou and Leu, 2010; Greiner *et al*., 2011; Barnard-Kubow *et al*., 2016). In the genus *Oenothera* subsect. *Oenothera* (Onagraceae), native to North America, three different nuclear genomes and five plastome types present in different combinations have been identified in various species. While the species readily form interspecific hybrids, only seven of the possible genome-plastome combinations produce plants with normal phenotypes and are commonly found in nature. The others show various degrees of cytonuclear incompatibility and only occur in nature as rare individuals. It seems that the limited number of compatible genome-plastome combinations maintain hybrid zones and species barriers across the geographical distribution of *Oenothera* (Dietrich *et al*., 1997; Greiner *et al*., 2011).

The strength of cytonuclear incompatibilities is likely to positively correlate with the rate of organelle evolution. The slow rate of sequence evolution in plant mitochondria and chloroplasts, compared to other eukaryotes (Wolfe *et al*., 1987; Clegg *et al*., 1994), potentially makes cytonuclear incompatibility less frequent in plants than in other organisms. However, cases of fast organelle evolution do exist in a number of plant species, and these may be linked to the adaptation of organelles to different ecological environments, potentially representing cases of co-evolution within populations (Sloan *et al*., 2012; Burton *et al*., 2013; Bock *et al*., 2014; Havird *et al*., 2017; Zwonitzer *et al*., 2024). In the cases of rapid organellar genome evolution, cytonuclear incompatibilities are common, and post-zygotic reproductive isolation often develops, creating hybridization barriers. In *Campanulastrum americanum* (Campanulaceae), one of the species with a rapidly evolving plastid genome, inter-population hybrids display a range of chlorotic phenotypes, with reduced survival of the F_1_ generation and substantial reproductive isolation dependent on the genetic divergence of the populations and the direction of the cross (Galloway and Etterson, 2005; Barnard-Kubow *et al*., 2014). Adaptation of cytoplasmic genomes to varying ecological environments and consequent species diversification has also been implicated in sunflowers (*Helianthus* sp.). Alloplasmic lines of mesic *H. annuus* (Asteraceae) and xeric *H. petiolaris* showed strong local adaptation of the cytoplasm of a given species to different habitats, which significantly affected the fitness of the hybrids (Sambatti *et al*., 2008). The effects of cytonuclear incompatibilities on phenotypes and ecology of hybrids were also revealed in other studies (Lee-Yaw *et al*., 2019; Thompson *et al*., 2024; Owens *et al*., 2025).

Cytonuclear incompatibility and its role in speciation may be further influenced by the inheritance pattern of organelles. Although maternal inheritance is predominant in angiosperms, approximately 20% of species have the potential to inherit organelles biparentally. In such cases, biparental inheritance may be selected for to mitigate the impact of incompatibility (Postel and Touzet, 2020). For instance, the F_2_ generation of *C. americanum* showed frequent recovery from the defective phenotype present in the F_1_ due to the switch to biparental inheritance of chloroplasts (Etterson *et al*., 2007; Barnard-Kubow *et al*., 2017).

Taken together, cytonuclear incompatibilities in plant hybrids often have strong deleterious effects on plant viability and fitness. However, these effects are frequently mitigated by various mechanisms (Box 1). This intricate interplay creates a delicate balance that allows establishment of hybrid lineages, potentially leading to plant speciation (Postel and Touzet, 2020).

Box 1. **How do plants restore/maintain cytonuclear compatibility?**

**Table:** 

Mechanism	Restores/maintains cytonuclear compatibility by …	References
*Genetic*		
Maternal homoeolog expression bias	Preferentially expressing maternally derived alleles to only include coevolved interacting subunits in cytonuclear enzyme complexes	Nomaguchi *et al*. (2018); Ferreira De Carvalho *et al*. (2019); Matos *et al*. (2019); Li *et al*. (2021, 2022); Zhang *et al*. (2023); Katayama *et al*. (2024); Zhao *et al*. (2025)
Maternally biased gene conversion, recombination, or segregation	Replacing paternal alleles with maternal nuclear alleles in genes with plastid or mitochondrial functions	Gong *et al*. (2012, 2014); Li *et al*. (2021, 2022); Wu *et al*. (2021); Ortiz and Sharbrough (2024)
Expression level dominance	Maternal imprinting of cytonuclear genes	June *et al*. (2024); Shahbazi *et al*. (2024)
Polyploidy	Maintaining diplo-sufficient levels of maternally derived cytonuclear genes via disomic inheritance of polyploid genome	Ferreira De Carvalho *et al*. (2019); Burns *et al*. (2021); Fernandes Gyorfy *et al*. (2021); Li *et al*. (2021); Forsythe *et al*. (2022); Grover *et al*. (2022); Hu *et al*. (2024); Ostovar *et al*. (2025); Prost-Boxoen *et al*. (2025); Shahbazi *et al*. (2025)
*Physiological*		
Increase in energetic capacity	Producing ‘enough’ energy to support cell/tissue/plant function/fitness by up-regulating cytonuclear enzyme complex expression and/or organelle biogenesis	Burns *et al*. (2021); Fernandes Gyorfy *et al*. (2021); Shahbazi *et al*. (2024, 2025); Ostovar *et al*. (2025)
Cell-type effects	Exhibiting greater maternal expression bias in energetically demanding/active cells than others	Zhang *et al*. (2023); Kan *et al*. (2024)
*Evolutionary*		
Loss/pseudogenization/silencing of paternally derived cytonuclear genes	Ensuring that only maternally derived alleles are present to only include coevolved interacting subunits in cytonuclear enzyme complexes	Sharbrough *et al*. (2022); Kan *et al*. (2024)
Expression level dominance	Selection favoring establishment of hybrids whose paternally derived subgenome has a greater degree of heterochromatin, DNA methylation, etc.	Yoo *et al*. (2014); Bird (2022); Kan *et al*. (2024)
Post-hybridization compensatory coevolution of nuclear and/or cytoplasmic genes	Evolving compensatory changes in nuclear or cytoplasmic genomes that allow for similarly successful interactions with either maternally or paternally derived nuclear alleles	Kan *et al*. (2024)
Loss of uniparental inheritance of organelles	(a) Ameliorating or (b) exacerbating incompatibilities between paternal nucleus and maternal cytoplasm resulting in selection (a) favoring or (b) preventing establishment of hybrids	a) Ramsey *et al*. (2019); Shrestha *et al*. (2021) b) Barnard-Kubow *et al*. (2017); Sobanski *et al*. (2019)

### Biased parental-specific retention of nuclear genes encoding organelle-targeted proteins in homoploid hybrids and allopolyploids

Cytonuclear incompatibility may arise between paternal variants of nuclear-encoded organelle-targeted proteins and organellar proteins (encoded by genes of maternal parent) due to divergence between the parental variants or their controlling *cis* and *trans*-acting factors. From an evolutionary perspective, and driven by positive selection, the most efficient way to restore functional interactions is to substitute paternal variants of the nuclear-encoded organelle-targeted genes with maternal variants (Hill *et al*., 2021). In principle, this can be achieved by segregation in the first meiotic division. In allopolyploids, homoeologous chromosome pairing is prerequisite for the segregation of two maternal alleles into a single gamete (Kopecký *et al*., 2008, 2009; Glombik *et al*., 2020). Alternatively, positive selection for amino acid substitutions in the paternal variant may also alleviate the mismatch (Gong *et al*., 2012, 2014; Li *et al*., 2020), although this does not appear to be particularly common in polyploids (Sharbrough *et al*., 2022). Another option is a complete excision of the paternal variants via gene loss or pseudogenization without the replacement by the maternal variant (Sharbrough *et al*., 2022; Sloan *et al*., 2024). However, this scenario presents a further source of disruption of cytonuclear interactions in homoploid hybrids—the altered proportion of the copy number of the nuclear vs. organellar genes involved in individual complexes in favor of the organellar genes. This phenomenon is observed in newly emerged autopolyploids, but in the opposite direction (nuclear genes are doubled after polyploidization; (Sharbrough *et al*., 2017)).

Contradictory results have been published in various taxa on the retention of maternal vs. paternal alleles of organelle-targeted nuclear genes in successive generations of allopolyploids. In the case of the RuBisCO complex, the nuclear-encoded gene *RBCS* has undergone paternal-to-maternal conversion in established allopolyploids such as *Arabidopsis suecica* (Brassicaceae), *Brassica napus* (Brassicaceae), *Arachis hypogaea* (Fabaceae), cotton (*Gossypium hirsutum*, Malvaceae), tobacco, and wheat (*Triticum aestivum*, Poaceae) (Gong *et al*., 2012, 2014; Li *et al*., 2020). In contrast, such conversion was not observed in rather young allotetraploid *Tragopogon mirus* (Asteraceae) and a synthetic allotetraploid rice (*Oryza sativa*) (Sehrish *et al*., 2015; Wang *et al*., 2017). Synthetic allopolyploid *Cucumis ×hytivus* (Cucurbitaceae) displayed unexpected maternal to paternal conversion (Zhai *et al*., 2019). An expanded analysis including many more cytonuclear systems was conducted by Ferreira De Carvalho *et al*. (2019) who studied the retention of parental alleles of over 100 genes involved in the cytonuclear complexes in allotetraploid *Brassica napus*. There was no clear evidence of maternally biased retention and gene expression. Using a genome-wide approach, molecular evolution of organelle-targeted genes in six allopolyploids including quinoa (*Chenopodium quinoa*, Amaranthaceae), coffee (*Coffea arabica*, Rubiaceae), cotton, wheat, tobacco, and *Brachypodium* (Poaceae) with different evolutionary histories showed a different pattern of parental-specific retention of organelle-targeted nuclear genes in each of the allopolyploids (Sharbrough *et al*., 2022). In general, the genes involved were less biased across subgenomes than the rest of the genome. Overall, it seems that the cytonuclear dynamics are highly taxon- and complex-specific. Each allopolyploid and each complex display different evolutionary patterns and there is no clear prominence of biased retention of parental alleles.

In terms of sequence evolution, rates of the protein/nucleotide evolution in paternal homoeologs of organelle-targeted genes are generally not increased (Sharbrough *et al*., 2022). In some allopolyploids, there is a difference between the two organelles: while the plastid-targeted nuclear genes display paternal-to-maternal switch, the mitochondria-targeted nuclear genes do not appear to experience homoeologous recombination in either direction (Ortiz and Sharbrough, 2024). To make it even more complicated, the retention of a parental allele might be, at least in some allopolyploids and homoploid hybrids, affected by the genome-wide dominance of one of the parents (sometimes called subgenome dominance; (Yoo *et al*., 2014; Alger and Edger, 2020; Glombik *et al*., 2021). In allotetraploid *Brassica carinata* (*B. nigra×B. oleracea*) and *B. juncea* (*B. rapa×B. nigra*), there is always reduced retention of the plastid-targeted genes encoded by *B. nigra* subgenome, regardless of whether *B. nigra* was the maternal or paternal donor (Kan *et al*., 2024). The subgenome of *B. nigra* displayed a higher abundance of transposons and other repetitive sequences compared to the genome of the other parent in both *B. carinata* and *B. juncea* (Kang *et al*., 2021; Yim *et al*., 2022). This supports the hypothesis that subgenome dominance can effectively drive evolutionary trajectories of cytonuclear interactions (Kan *et al*., 2024).

In addition to protein-coding genes, nuclear-encoded chaperonins and chaperones involved in folding and assembly of protein complexes are of particular interest. If the two parental variants differ at the amino acid level (in a functional domain), they may also cause cytonuclear incompatibility. However, little is known about the retention and expression of the parental forms of chaperonins and chaperones. Li and colleagues (Li *et al*., 2022) found a frequent paternal-to-maternal homoeologous gene conversion of the chaperonins and chaperones of RuBisCO in four different allopolyploids (wheat, cotton, tobacco and *Arabidopsis*), indicating similar evolutionary genomic and transcriptomic patterns as the nuclear-encoded gene (*RBCS*). However, this response, together with the maternal homoeolog expression bias (HEB), was temporally attenuated, or even diminished, during folding and later assembly processes. This analysis revealed that chaperonins involved in protein folding might be more inclined toward cytonuclear coordination than chaperones operating later in protein assemblies. Gene conversion of chaperonins and chaperones was frequently different in synthetically derived allopolyploids compared to their natural allopolyploid counterparts, with occasional examples of maternal-to-paternal conversion, while transcription trends were generally highly similar (Li *et al*., 2022). It appears that paternal-to-maternal gene conversion or homoeologous exchange is rare in the first generation after hybridization, and that these processes require one or more generations (i.e. at least one meiotic cycle; (Lindegren, 1953); (Daugherty and Zanders, 2019)) to become consequential. Thus, other mechanisms may be involved in the initial responses to cytonuclear incompatibilities. Most prominently, biased transcription of maternal variants has come to the forefront in allopolyploids and homoploid hybrids, as discussed in the next section.

### Parent-specific expression of nucleus-encoded genes involved in cytonuclear complexes

The presence of an incompatible, paternally derived nuclear allele, even in heterozygous form, can impair multi-subunit complex assembly dynamics, substrate binding affinity, or other functions. Depending on the degree to which the presence of such an allele reduces organismal energy budgets and/or homeostasis, or causes oxidative damage due to the production of excessive reactive oxygen species (ROS) in energetically demanding cells or tissues, homoeolog-specific expression regulation could help improve the mean enzyme activity and potentially reduce ROS production. Bulk-tissue RNA sequencing has generally failed to find evidence related to the maternally biased gene expression of cytonuclear enzyme complex genes after accounting for the expression-level dominance (ELD) and the parental legacy effects (Grover *et al*., 2022). However, recent work employed single-cell RNA sequencing (scRNAseq) in allotetraploid cotton, allotetraploid peanut (*Arachis hypogaea* L.), and allohexaploid wheat that revealing that photosynthetically active cells exhibit a maternal HEB in the nuclear-encoded genes involved in plastid-nuclear enzyme complex (Zhang *et al*., 2023). Such a pattern may also be present in other allopolyploids, but the extent to which this is true must await additional scRNAseq data in other taxa and tissues.

Various mechanisms that can generate maternal HEB of the cytonuclear enzyme complex genes are still being elucidated; however, we may speculate on some possibilities. In principle, maternal expression bias can be achieved at several distinct regulatory points: pre-transcriptional, co-transcriptional, post-transcriptional, protein import, enzyme complex assembly, proteasome degradation, or any combination thereof. The pre-transcriptional maternal HEB (i.e. expression bias beyond ELD acting through global biases across subgenomes in heterochromatin or DNA methylation) would likely require a retrograde signal, such as a cytoplasmically encoded gene product, which could function as a specific transcription factor. This hypothetical regulator would have coevolved with the maternal subgenome, so it could be capable of differentiating between the enhancers of the maternal vs. paternal genomes. ChIP-seq (Chromatin Immunoprecipitation Sequencing) or ATACseq (Assay for Transposase-Accessible Chromatin with sequencing) paired with RNAseq datasets have proliferated in a number of different taxa in recent years (Hu *et al*., 2024), allowing for a targeted analysis of biases in the cytonuclear enzyme complex genes in maternal vs. paternal genomes. Co-transcriptional regulation could act via biased intron retention across homoeologs (de Jong and Adams, 2023), which prevents mRNAs with unspliced introns from leaving the nucleus, and also appears to be affected by the presence of specific transcription factors (Ullah *et al*., 2023). Maternal HEB in intron retention (or other alternative splicing regulatory mechanisms) could thus be assessed using datasets of paired long-read transcriptomes with quantitative RNAseq. Unlike pre- or co-transcriptional regulation, differential post-transcriptional regulation of maternal vs. paternal transcripts would likely require post-hybridization time to evolve, as the paternal subgenome would need to accumulate homoeolog-specific miRNAs that could act to reduce the translation rates of the paternally derived transcripts. Analysis of microRNA (miRNA) abundance and diversity in allopolyploid genomes would provide fascinating evidence bearing on how cells regulate homoeologous alleles. Besides regulation via miRNAs, hybrids might translate parental alleles with a different efficiency. Differences in translational efficiency between parental variants appeared to buffer the transcriptional differences in hybrid rice (Xi *et al*., 2025).

Although the chloroplast and mitochondrial import machinery is entirely nuclear-encoded, and should therefore not be biased, maternal bias has nevertheless been demonstrated in hexaploid wheat (Li *et al*., 2020). Here, the target peptides of the RuBisCO SSU protein encoded by the D-genome (paternal genome) have experienced homoeologous conversion to resemble the B-genome target peptide and later concerted evolution between *RBCS* gene copies. This appears to have resulted in the down-regulation of the D-genome’s copy of the TOC90 protein, which recognizes the D-genome chloroplast transit peptide. The result is that protein accumulation in the chloroplast is expected to be biased in favor of the A- and B-genomes. At the complex assembly level, if the paternally derived protein subunits cause delays in the enzyme complex assembly, maternal HEB may be unbiased at the transcript level, but highly biased at the protein-complex level. Finally, an effective maternal HEB could be established by biased enzyme complex degradation via the proteasome. If, for example, complexes that contain paternally derived subunits produce higher levels of ROS (Yamamoto *et al*., 2008), they may be selectively degraded at a faster rate than the entirely maternally derived protein complexes. Development of methods that can distinguish between alleles or homoeologs at the protein level, and quantified precisely, will be critical for evaluating the last two possibilities.

In summary, there are numerous possibilities for how maternal HEB can be established for cytonuclear genes specifically, but all fundamentally require that (1) there are functional differences in cytoplasmically encoded genes and gene products, and that (2) the expression and inclusion of paternally derived transcripts or proteins negatively impact energy budgets and/or exposure to oxidative damage. Importantly, cells and tissues are capable of buffering these negative consequences via physiological changes, which makes it particularly difficult to assess whether enzyme complex performance is in fact reduced.

### Cytoplasm-to-organelle protein trafficking

The nuclear-encoded proteome of organelles in hybrid cells is shaped not only by allele-specific expression, homeolog expression bias, and protein translation efficiency, but also by allele-specific organelle targeting and import (Agrawal *et al*., 2011; Grover *et al*., 2012). Nuclear-encoded cytonuclear subunits destined for chloroplasts or mitochondria traverse multi-step pathways from the cytosol to the organelle. Sequence divergence can affect several steps of this journey and thereby modulate import throughput (Thagun *et al*., 2024; Rödl *et al*., 2025). These processes rely on the N-terminal targeting signals, together with guidance/holding factors and import machineries located in the organelle membranes (May and Soll, 2000; Young *et al*., 2003; Araiso *et al*., 2022; Liang *et al*., 2024).

Chloroplast transit peptides (cTPs) are intrinsically disordered, Serine/Threonine-rich, depleted in acidic residues, and typically Arginine-poor near the extreme N-terminus, properties enabling efficient recognition by the plastid TOC/TIC import machinery (Jeong *et al*., 2021; Ballabani *et al*., 2023). By contrast, mitochondrial targeting peptides (mTPs) form N-terminal, positively charged amphipathic α-helices, usually ∼35 amino acids long but sometimes spanning ∼10–120 amino acids (Von Heijne, 1986). Because cTPs are compositionally flexible, they often tolerate sequence variation, though cross-genome interchangeability depends on species-specific receptors. In contrast, mTPs require a conserved amphipathic, basic helix for TOM (translocon at the outer mitochondria membrane)/TIM (translocon at the inner mitochondria) membrane recognition, probably constraining interchangeability (Murcha *et al*., 2014; Almagro Armenteros *et al*., 2019). Several studies have revealed that even small changes in presequences (short N-terminal extensions that direct nuclear-encoded proteins to organelles) can shift the preprotein targeting and translocation efficiencies. For example, replacing Lysines with Arginines in targeting signals improved targeting to both organelles, underscoring how alleles might differ in import preference (Caspari *et al*., 2023; Thagun *et al*., 2024; Rödl *et al*., 2025).

Considering the concept of strong versus weak targeting signals, preprotein import into organelles is best framed in quantitative terms, such as maximal velocity, and prioritization under allelic competition for a limited pool of the translocators (Rödl *et al*., 2025). In mitochondria, specific combinations of amino acids in the presequences were shown to confer different import efficiencies among co-expressed precursors (Rödl *et al*., 2025), indicating that presequences vary in their ability to direct proteins into the organelle with different levels of efficiency (Yan *et al*., 2025, Preprint). Collectively, systematic screens and motif manipulations demonstrate that even small changes in targeting signals can markedly shift import probabilities (Thagun *et al*., 2024; Boob *et al*., 2025). Direct quantitative tests of the allele-specific import competition in plant hybrids (e.g. using different fluorescent tags for both homoeologs) remain an important target for future work.

Following interspecific hybridization or allopolyploidization, parental alleles of the organelle-targeted genes frequently encode divergent transit peptides. Such differences can alter recognition by plastid (TOC/TIC) or mitochondrial (TOM/TIM) receptors, potentially biasing import when homoeologs are co-expressed and compete for a limited import capacity. In chloroplasts, diversified TOC receptors (Toc159/132/120 with Toc33/34) display substrate preferences linked to cTP features and the developmental state, and their activities are further tuned by post-translational regulation. In particular, phosphorylation-dependent control has been demonstrated for TOC33, adding a regulatory layer to isoform specialization (Kubis *et al*., 2004; Demarsy *et al*., 2014; Chien and Yoon, 2025). Recent analysis of the structure and regulation emphasizes that import is not static. TOC/TIC architecture, the AAA+Ycf2-FtsHi (ATPases Associated with diverse cellular Activities–type Ycf2–FtsHi chloroplast protein import motor complex) motor, and Ubiquitin–Proteasome System (UPS)-linked turnover have been further resolved, refining where sequence variants might alter the flux; at the same time, aspects of plastid inner-envelope composition remain unknown (Liang *et al*., 2024).

Protein import into the chloroplast is regulated at several checkpoints: pathway surveillance mechanisms, Chloroplast-Associated Degradation(CHLORAD)-mediated ubiquitination and retrotranslocation of TOC subunits, and stress-induced selective autophagy that removes import hubs. These nodes can, in principle, amplify or buffer allele-specific differences in import efficiency. When misregulated, they lead to dosage imbalances, proteostatic stress, and growth defects (Li and Jarvis, 2024; Hong *et al*., 2025). In mitochondria, TOM20 and TOM22 recognize canonical presequence motifs with defined amphipathic and charge patterns, establishing another layer at which alleles from two divergent genomes can differ in receptor engagement and import rate (Abe *et al*., 2000; Saitoh *et al*., 2007; Yamano *et al*., 2008; Rimmer *et al*., 2011). By contrast, upstream cytosolic holdases (e.g. 14-3-3 dimers and Heat shock protein (Hsp) 70s) that keep precursors in an unfolded state (therefore facilitating their translocation) are widely conserved across eukaryotes and therefore probably impose fewer allele-specific recognition rules than organelle receptors, although they still modulate the flux and can shape the precursor fate (Obšilová *et al*., 2008; Craig, 2018). Thus, the most plausible sources of allelic competition and BDM incompatibilities at the stage of targeting of preproteins to the organelles are (1) divergence in the signals on the preproteins themselves and (2) divergence or regulatory reconfiguration of the organelle receptors that read those signals. As mentioned earlier, the paternal D-genome *RBCS* allele in hexaploid bread wheat initially encoded a transit peptide poorly matched to the maternal A/B-genome derived chloroplast import machinery, likely reducing import efficiency. A paternal-to-maternal gene-conversion event replaced part of the transit-peptide coding region of the D allele with the B-genome sequence, effectively maternalizing the signal and restoring compatibility with the hybrid’s TOC receptors (Li *et al*., 2022).

Overall, hybrid contexts expose fine-scale differences in targeting signals and receptor repertoires. Where one allele’s chloroplast (or mitochondria) transit peptide better matches the resident receptor landscape, or encodes a higher-priority import signature, it is expected to gain preferential translocon access, generating allele-specific import bias and setting the stage for adaptive editing (conversion) or selection on targeting sequences in hybrid genomes. Functionally, such bias can cascade through altered complex stoichiometry, compensatory transcription/translation responses, excessive cytosolic precursor load, activation of chloroplast import quality-control, and shifts in the organelle proteome composition, with measurable consequences for photosynthesis, respiration, and hybrid vigor or breakdown (Postel *et al*., 2024; Thagun *et al*., 2024).

### Protein folding and assembly assisted by chaperonins and chaperones

Once imported into chloroplasts or mitochondria, precursor proteins must fold correctly and assemble into multi-subunit complexes to become functional. This maturation depends on numerous molecular chaperones and assembly factors, almost all of which are nuclear-encoded in plants (Fox, 2012; Vitlin Gruber and Feiz, 2018). The nuclear origin of this folding machinery implies that it coevolved with both nuclear- and organelle-encoded interaction partners (Kessler and Schnell, 2009; Jarvis and López-Juez, 2013). Hybridization can therefore disrupt this co-adaptation and generate the BDM incompatibilities at folding and assembly stages, with measurable fitness costs (Barnard-Kubow *et al*., 2016; Sloan *et al*., 2018; Li *et al*., 2022). Misfolded subunits or inefficient complexes can elevate ROS, activating plastid-to-nucleus retrograde signaling that reprograms nuclear transcription and can even alter chromatin configuration (Kleine and Leister, 2016; Quevedo *et al*., 2025). In the chloroplast stroma, cpHsp70-family chaperones bind newly imported polypeptides to suppress aggregation and promote productive folding; cpHsp70 acts at the import translocon and during early folding steps in parallel with Hsp93/Tic40 (Shi and Theg, 2010). In the mitochondrial matrix, Hsp70 and the chaperonin system similarly guide post-import maturation.

Chaperonins (Cpn) use ATP to form a closed space where proteins can undergo folding. In chloroplasts, Cpn60 works with Cpn20/Cpn10, while in mitochondria, Hsp60 works with Hsp10. These systems prevent misfolding by holding proteins inside their cavity (Zhao and Liu, 2018). Consistent with this, multiple studies emphasize the essentiality of Cpn60/Cpn20 for plastid proteostasis and for assembly of highly abundant clients such as RuBisCO (Vitlin Gruber and Feiz, 2018; Zhao and Liu, 2018). RuBisCO biogenesis illustrates how sequence differences at the client-chaperone interfaces can shape the outcomes of protein folding. The LSU of RuBisCO requires Cpn60/Cpn20 for folding, followed by assembly with auxiliary factors RuBisCO assembly factors 1 and 2, RuBisCO binding chaperone X (RbcX) and Bundle Sheath Defective 2 (BSD2) (Feiz *et al*., 2014; Aigner *et al*., 2017; Bracher *et al*., 2017; Vitlin Gruber and Feiz, 2018). Heterologous expression of RuBisCO subunits makes this specificity visible: *Arabidopsis* RuBisCO can only assemble in *E. coli* when the cognate chloroplast chaperonin, plus the full auxiliary set, are co-expressed, and yields improve when expression is carefully tuned (Aigner *et al*., 2017; Wilson *et al*., 2019). The hornwort (*Anthoceros agrestis*, Anthocerotaceae) RuBisCO requires hornwort-specific chaperones for complete biogenesis in a heterologous system, underlining client-chaperone coevolution (Oh *et al*., 2024). Mapping the protein contact surfaces shows that proper assembly depends on specific and selective contacts: in RuBisCO, Raf1 recognizes the LSU βC-βD loop as a key determinant, but only from phylogenetically related species and not from more distant species, helping explain why non-cognate combinations often fail (Archer *et al*., 2025). Functionally, these rules scale to growth: transgenic co-expression of LSU/SSU with Raf1 or Raf2 can accelerate plant growth, whereas mis-matched auxiliary sets reduce accumulation of the functional protein complexes (Huang *et al*., 2020; Eshenour *et al*., 2024). Plant genomes encode additional paralogs (e.g. RafL) that dimerize with Raf1 to modulate the assembly trajectories, adding another potential axis for the allele-specific effects in hybrids (Cheng *et al*., 2024).

Taken together, available evidence indicates that moderate sequence divergence at the client-chaperone contact surfaces can, in some cases, derail folding checkpoints and stabilization, producing low levels of RuBisCO (or other cytonuclear enzyme complexes), chlorosis, and fitness costs in hybrids; matched pairs restore biogenesis (Aigner *et al*., 2017; Archer *et al*., 2025). This helps explain why chaperones and assembly factors are the frequent targets of gene conversion and gene expression modification after interspecific hybridization. In line with the cytonuclear co-evolution hypothesis, some RuBisCO folding/assembly networks show bias toward the maternal homoeologs in allopolyploids, consistent with selection to match the maternally inherited organelle system. A comparative multi-lineage analysis reported paternal-to-maternal gene conversions and maternal HEB in chaperonin/chaperone genes, assisting RuBisCO biogenesis, with stronger signals at early folding steps (Li *et al*., 2022). Plant reproduction is likewise shaped by co-evolution between nuclear and organelle genomes under predominantly maternal cytoplasmic inheritance (Kan *et al*., 2024; Chung, 2025). Given the specific coevolutionary history required for cytonuclear enzyme complex assembly in each natural system, understanding how complex assembly proceeds and contributes to variation in fitness in natural hybrids represents an important future research direction that promises to shed light on cytonuclear mechanisms of adaptation and diversification.

### Cytoplasmic male sterility and its practical applications

Although cytonuclear incompatibilities are most often associated with negative fitness consequences, they may also yield traits of considerable practical value. Cytoplasmic male sterility (CMS) represents a specific manifestation of cytonuclear incompatibility, expressed during male gametophyte development, in which mitochondrial-encoded sterility factors interfere with pollen development in hermaphroditic plants unless counteracted by compatible nuclear genes (Carlsson *et al*., 2008; Tang *et al*., 2017). CMS has therefore become a widely used tool in hybrid crop breeding.

Implementation of hybrid breeding in self-compatible species (e.g. maize and rice) ranks highly among the key factors contributing to improvements in these crops, starting in the 1960s. However, the laborious manual de-tasseling to remove male parts so that the advantages of hybridity were retained remained a substantial shortcoming. This impediment was overcome by the identification and harnessing of self-incompatibility systems, usually via CMS (Chen and Liu, 2014). The result is a male-sterile, female-fertile plant incapable of selfing. Successful deployment of CMS has aided in the development of hybrid CMS cultivars in a variety of crops. To our knowledge, the first commercialization of hybrid rice in China is dated to 1975 and rice yields increased by 44% between 1978 and 2008 due, at least in part, to the incorporation of hybrid rice varieties featuring CMS into agricultural practice (Li *et al*., 2009; Chen and Liu, 2014). Even greater improvements were achieved in maize, where introduction of hybrids using CMS led to the enormous yield increase over last six decades, from 40 to 180 bushels per acre (Tranel, 2024).

**Fig. 2.**
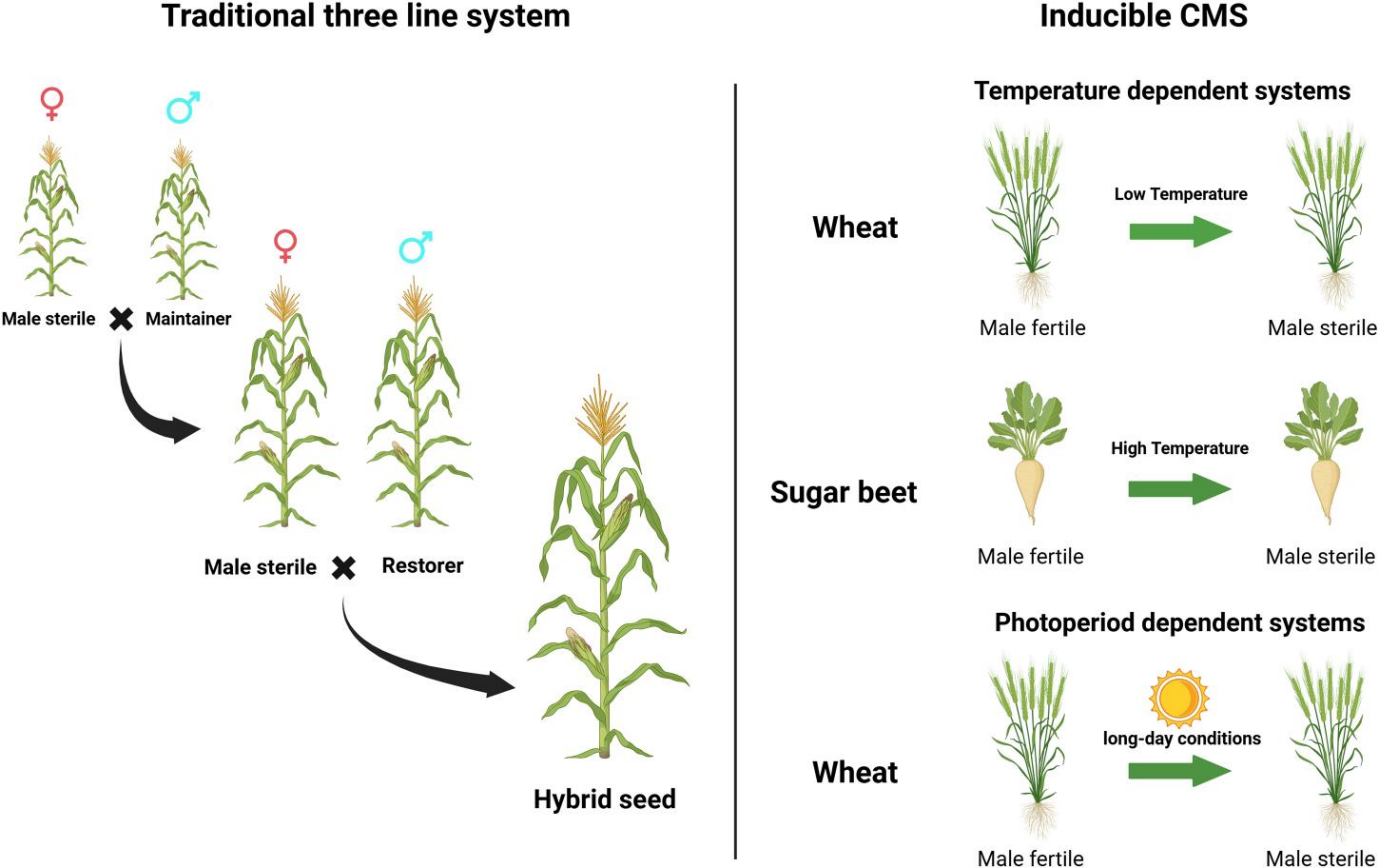
Cytoplasmic male sterility (CMS) in crop breeding programs. A specific form of cytonuclear incompatibility is CMS, which is expressed during male gametophyte development and results from organellar-encoded genes (most commonly mitochondrial) that disrupt pollen formation in otherwise hermaphroditic plants. The traditional three-line system (left panel) explains the crossing scheme maintaining CMS (male sterile) plants and producing hybrids with high heterosis using a restorer line as the pollinator. Environmental conditions may trigger induction of CMS (right panel). Created in BioRender. Kopecky, D. (2026) https://BioRender.com/rrm39jo.

CMS-based breeding technology relies on a three-line system: the CMS line contains the sterility gene in the mitochondrial DNA but lacks the nuclear *Rf* (*Restorer-of-fertility*) gene that normally restores fertility (Melonek *et al*., 2021); the maintainer line also lacks the *Rf* gene but has a male-fertile cytoplasmic genome; and the restorer line contains a functional *Rf* allele (Fig. 2, left). The CMS line is always used as the female parent. The maintainer serves as the male parent to perpetuate the CMS line, while the restorer line is used as male parent to generate the hybrid with heterosis (Chen and Liu, 2014). Numerous different CMS systems have been reported in crucifers, among others, including Ogura CMS (Ogu CMS) identified in *Raphanus sativus* (Brassicaceae), *Brassica napus*, *B. rapa*, *B. oleracea,* and several other species (Ren *et al*., 2022).

Besides utilization of the CMS approach in intraspecific hybrid breeding and seed production (such as maize and rice), CMS was also observed in alloplasmic lines with a nuclear genome inherited from one species and cytoplasm, including plastids and mitochondria, from another species. The resulting disruption to cytonuclear interactions causes pollen abortion, and thus male sterility, without affecting female gametes in F_1_ hybrids (Kaminski *et al*., 2012; Yamagishi and Bhat, 2014). Bulb onion (*Allium cepa* L.) breeding is based entirely on hybrid seed production because of its seed-propagation capacity (Shigyo and Kik, 2008) and thus, male sterility is one of the most important characteristics in the breeding of *Allium* (Amaryllidaceae) crops (Brewster, 2008). CMS lines are obtained by crosses of cultivated *A. cepa* with wild relative *A. royei,* followed by several backcrosses to *A. cepa*, thus combining the cytoplasm of one species with the nuclear genome of the other (Vu *et al*., 2011). Besides maintaining heterosis via CMS, alloplasmic lines may offer other profitable traits for crop improvement. The cytoplasm of *Aegilops kotschyi* (Poaceae) in alloplasmic lines of bread wheat causes not only male sterility, but also affects growth rate, pollen competition for fertilization, early endosperm divisions, seed size and plant maturity (Fan *et al*., 2024), and perhaps tolerance to abiotic stresses (Bocianowski and Prażak, 2022). Moreover, the combination of the *Ae. kotschyi* cytoplasm and a rye (*Secale cereale* L.) 1RS inducer is well known for haploid production (Tsunewaki *et al*., 1974). The potential of alloplasmic lines for crop improvement is also evident in citrus (*Citrus* spp.), chicory (*Cichorium intybus* L.), rucola (*Eruca sativa* Mill.), sugar beet (*Beta vulgaris* L.), eggplant (*Solanum melongena* L.), sunflower (*Helianthus annuus* L.) and other crops (Chepurnaya *et al*., 2003; Yamagishi and Bhat, 2014; Bohra *et al*., 2016; Faddetta *et al*., 2018).

Recent technological advancements have enabled a more targeted approach for implementing CMS in crop improvement efforts. Long-read sequencing has produced whole genome assemblies in many crops, a resource that & is often enhanced by the generation of pan-genomes and pan-plastomes from the sequencing of multiple accessions (reviewed in (Hu *et al*., 2024)). This has significantly increased our understanding of the molecular mechanisms underlying cytonuclear incompatibility and CMS. It has also simplified the selection of causative candidate genes, even among crop wild relatives. Complete organelle genome sequences have enhanced the development of DNA markers used to identify CMS in marker-assisted selection (Choe *et al*., 2023; Farinati *et al*., 2023). Novel breeding approaches to CMS take advantage of the environmentally dependent responses of certain CMS and *Rf* alleles (Fig. 2, right). Environmentally unstable CMS, such as temperature-dependent systems, can be used as inducible CMS if the conditions are precisely defined (Kitazaki *et al*., 2023). Temperature-dependent *Rf* alleles have been used to generate thermosensitive CMS lines. For example, the recessive nuclear gene rfv_1_^m^ (restoring fertility v1) from wild relative *Triticum macha* has been exploited in wheat breeding to induce male sterility at low temperatures (Meng *et al*., 2016). CMS can also be induced by high temperatures, as in the case of sugar beet (Matsuhira *et al*., 2022). Photoperiod sensitive CMS lines have also been generated in wheat. These lines are male sterile under long-day conditions but fertile under short-day conditions (Murai *et al*., 2008, 2016). Although genome editing, specifically CRISPR/Cas9 technology, is a popular approach for precise modification of the plant genome, it has not yet been used to generate CMS lines because mitochondrial DNA editing still represents a significant challenge. However, CRISPR-assisted development of male-sterile mutants have been achieved by knocking out nuclear *Rf* genes (Qi *et al*., 2020; Farinati *et al*., 2023) representing an alternative path for breeding programs.

The above synopsis demonstrates the immense agricultural importance of CMS in numerous crop plants, with the corollary that developing a deeper mechanistic understanding of the many processes and pathways involved in CMS would be of further benefit to agriculture. This understanding is likely to derive from integrative approaches from multiple crop systems as well as application of the full spectrum of molecular biological and genomic technologies.

## Summary and future progress

Cytonuclear interactions represent a complex suite of biological and physiological processes at the molecular level which are important in hybridization and diversification in natural populations as well as in targeted crop-improvement efforts. The specific and complex interactions between mitochondrial-, plastid-, and nuclear-encoded genes and gene products set the stage for numerous levels of biological regulation, and potential for manipulation. In particular, hybrids must reconcile a nuclear genome inherited from two different species but cytoplasmic genomes from a single species, such that interactions between genomic compartments are likely to affect hybrid lineage function and evolutionary outcomes. Successful hybrids, both diploid and polyploid, are known to implement various genetic, physiological, and evolutionary mechanisms that aid in the amelioration of mismatches between the nuclear and cytoplasmic genomes. Restoration of cytonuclear compatibility in hybrid lineages via allele-specific responses can thus proceed at multiple levels of regulation, including DNA methylation/chromatin accessibility, gene expression, alternative splicing, translation rates, organelle import, protein-folding and assembly, and protein degradation pathways. Most of these potential molecular mechanisms have only been investigated with the use of bulk-tissue RNAseq, so spatial and/or single-cell transcriptomics, high-resolution protein sequencing and quantification, fluorescent microscopy, and the slew of modern molecular biology tools will be critical in documenting the molecular activity of mismatched cytonuclear combinations. Considering the importance of hybridization for plant speciation, diversification, and domestication, investigating each of these molecular mechanisms will provide important insights into plant biology. This work has the potential to be particularly impactful for targeted crop improvement efforts—by leveraging CMS we can establish and maintain superior hybrid lineages.

## Abbreviations

BDM incompatibility: Bateson-Dobzhansky-Muller incompatibility
CMS: cytoplasmic male sterility
cTPs: chloroplast transit peptides
ELD: expression level dominance
HEB: homoeolog expression bias
LSU: large subunit of RuBisCO
miRNA: micro RNA
mTPs: mitochondrial targeting peptides
OXPHOS: Oxidative phosphorylation
*RBCS*: nuclear gene encoding SSU
*Rf*: *Restorer-of-fertility* gene
ROS: reactive oxygen species
RuBisCO: Ribulose-1,5-bisphosphate carboxylase/oxygenase
scRNAseq: single-cell RNA sequencing
SSU: small subunit of RuBisCO
TIC: translocon at the inner chloroplast membrane
TIM: translocon at the inner mitochondria
TOC: translocon at the outer chloroplast membrane
TOM: translocon at the outer mitochondria membrane
